# A More Desirable Balanced Polyunsaturated Fatty Acid Composition Achieved by Heterologous Expression of Δ15/Δ4 Desaturases in Mammalian Cells

**DOI:** 10.1371/journal.pone.0084871

**Published:** 2013-12-31

**Authors:** Guiming Zhu, Qin Ou, Tao Zhang, Xudong Jiang, Guozhi Sun, Ning Zhang, Kunfu Wang, Heng Fang, Mingfu Wang, Jie Sun, Tangdong Ge

**Affiliations:** 1 Laboratory of Biochemistry and Molecular Biology, College of Basic Medicine, Jiamusi University, Jiamusi, Heilongjiang, China; 2 Jiamusi College, Heilongjiang University of Chinese Medicine, Jiamusi, Heilongjiang, China; 3 College of Pharmacy, Jiamusi University, Jiamusi, Heilongjiang, China; National Institute of Nutrition, India

## Abstract

Arachidonic (ARA), eicosapentaenoic (EPA) and docosahexaenoic (DHA) acids are the most biologically active polyunsaturated fatty acids, but their biosyntheses in mammals are very limited. The biosynthesis of DHA is the most difficult, because this undergoes the Sprecher pathway–a further elongation step from docosapentaenoic acid (DPA), a Δ6-desaturase acting on a C24 fatty acid substrate followed by a peroxisomal chain shortening step. This paper reports the successful heterologous expression of two non-mammalian genes (with modification of codon usage), coding for *Euglena gracilis* Δ4-desaturase and *Siganus canaliculatus* Δ4-desaturase respectively, in mammalian cells (HEK293 cell line). Both of the Δ4-desaturases can efficiently function, directly converting DPA into DHA. Moreover, the cooperation of the *E. gracilis* Δ4-desaturase with *C. elegans* Δ15-desaturase (able to convert a number of n-6 PUFAs to their corresponding n-3 PUFAs) in transgenic HEK293 cells made a more desirable fatty acid composition – a drastically reduced n-6/n-3 PUFAs ratio and a high level of DHA as well as EPA and ARA. Our findings provide a basis for potential applications of the gene constructs for expression of Δ15/Δ4-desaturases in transgenic livestock to produce such a fatty acid profile in the related products, which certainly will bring benefit to human health.

## Introduction

The importance of polyunsaturated fatty acids (PUFA) for human health has been well recognized. Among PUFAs, arachidonic (ARA; 20∶4n-6), eicosapentaenoic (EPA;20∶5n-3) and docosahexaenoic (DHA; 22∶6n-3) acids are the most biologically active essential fatty acids (EFA) in mammals. ARA can be synthesized by Δ6 desaturation of linoleic acid (LA;18∶2n-6) to produce 18∶3n-6 that is elongated to 20∶3n-6 followed by Δ5 desaturation [1], while EPA can be synthesized from a-linolenic acid (ALA,18∶3n-3), requiring the same enzymes and pathway as for ARA. Synthesis of DHA reportedly requires an elongation step from EPA to docosapentaenoic (DPA;22-5n-3, an important intermediate), a further elongation step to C24 PUFA, a second Δ6 desaturation and a peroxisomal chain shortening step (*β*-oxidation) [2]. Therefore, LA and ALA are considered essential fatty acids in the human diet. However, the rate of conversion of LA to ARA, or ALA to EPA and DHA, remains low [3]–[5]. The conversion of LA to ARA is ∼0.2%(6). A very small fraction (perhaps <1%) of ingested ALA is converted into EPA (20∶5n-3), and even less is converted into the 22-carbon DHA. Thus, these long-chain PUFA composition of cell membranes are, to a great extent, dependent on dietary intake [7], [8]. On the other hand, natural resources for n-6 PUFAs (such as LA and ARA) and n-3 PUFAs (such as ALA, EPA and DHA) are imbalanced, leading to unhealthy diets with a high ratio (∼10∶1) of n-6/n-3 PUFAs [9], [10]. This may contribute to the prevalence of many diseases, such as coronary artery disease, cancer, diebetes, arthritis and depression [11], [12].

To increase the n-3 PUFA content and decrease the ratio of n-6/n-3 PUFAs in diet is conductive to human health. Hence efforts have been made to achieve this goal. A Δ15 (n-3) desaturase gene from *C. elegans*, *fat-1*, was introduced into mammalian cells then into animals such as mice and pigs, and consequently the desaturase produced n-3 PUFAs from the corresponding n-6 PUFAs in transfected mammalian cells and transgenic animals [13]–[16]. This is a major breakthrough in the research field of n-3 PUFAs production. However, *fat-1* gene mainly converted C_18_ and C_20_ n-6 PUFAs to their corresponding n-3 PUFAs (such as ALA, EPA, DPA) and increased their content, without or with very limited increase of DHA, the most important C_22_ PUFA. Previously, Δ4 desaturases have been demonstrated in some organisms including protozoan trypanosomes [17], the photosynthetic freshwater protist *Euglena gracilis* [18], marine microalgae *Pavlova lutheria* and *Thraustochytrids* [19], [20], and herbivorous, marine teleost fish, *Siganus canaliculatus* [21]. By heterologous expression in yeast, these Δ4 desaturases conferred on the yeast the ability to convert DPA (22∶5n-3) to DHA. We realized that if such a Δ4 desaturase gene can still function in mammals to convert DPA to DHA, it would coorperate with the Δ15 desaturase gene, *fat-1*, to achieve a more desirable fatty acid composition (balanced n-6/n-3 PUFAs with a high level of DHA).

However, whether these Δ4 desaturase genes can be expressed functionally and can coorperate with *fat-1* gene in mammalian cells remains to be investigated. According to the reports in the related literatures, we chose two Δ4 desaturase genes, one from *E. gracilis*, the other from *S. canaliculatus*, as the candidates in our research. The objective of this study was to examine whether the two Δ4 desaturase genes can be functionally expressed in mammalian cells in high efficiency and whether the Δ4 desaturase activities can coorperate with the Δ15 desaturase activities to exert a significant effect on cellular fatty acid composition.

## Materials and Methods

### Construction of Recombinant Plasmids

Both the gene sequences coding for *Euglena gracilis* Δ4 desaturase (named as *sEgD4* ) and *Siganus canaliculatus* Δ4 desaturase (named as *sScD4*), whose original sequences were submitted to GenBank (accession number AY278558 and GU594278, respectively ), were artificially synthesized (with genetic codons optimized for mammalian expression). Then the synthetics were separately inserted into the pcDNA3.1 expression vector by EcoR I/Hind III sites (both synthetics carrying the same enzymic sites). The *fat-1*gene (also synthesized with codons optimized, named as *sN3*) expression vector was constructed similarly as the two Δ4 desaturase gene expression vectors. The double gene expression vector pcDNA3.1-N3D4 was constructed as follows: making use of Bgl II/Mlu I sites carried on pcDNA3.1, PCR primers (with Bgl II or Mlu I site sequence on 5′end ) were designed to amplify the pCMV-sN3-polyA DNA fragment on pcDNA3.1-sN3, and then the amplicon was inserted into pcDNA3.1- sEgD4 by Bgl II/Mlu I double digestion and ligation.

### Cell Cultures and Transfection

Human embryonic kidney (HEK) 293 cell line was obtained from American Type Culture Collection (Manassas, VA, USA). Transient transfections in HEK 293 cells were carried out using Lipofectamine™ 2000 (Invitrogen Inc., USA) as described in the manufacturer’s manual. One day prior to transfection, cells were plated onto 60-mm culture dishes in Dulbecco’s modified Eagle’s medium (DMEM) supplemented with 10% fetal bovine serum (FBS) and 10 µM PUFAs substrates (DPA for Δ4 desaturase genes transfections, LA and ARA for *sN3* or *N3D4* transfections). Cells were transfected with 8 µg of the plasmids. To monitor transfection efficiencies, cells were transfected with an expression vector containing the enhanced green fluorescent protein (EGFP) cDNA, pcDNA3.1-EGFP. The day following transfection, the transfection medium was replaced with fresh medium containing the same PUFAs substrates as above. Two days after transfection, cells were harvested and used for analysis of gene expression or fatty acid composition.

### RT-PCR

RT-PCR was performed to analyze transcripts in transgenic cells.Total RNA was extracted from the harvested cells using TRIzol Reagent (Invitrogen) followed by DNase treatment to remove genomic DNA contamination. cDNA was prepared using the TaKaRa RNA PCR Kit 3.0 (TaKaRa). The following primer pairs were used to detect the target genes–*sEgD4*∶5′-TCATCATCAACCACATCAGCGAG-3′ and 5′-TTTAGCTCTTCTTGTCGCCGTTG-3′; *sScD4*∶5′-TCGGGCATCTGAGCGTGTTC-3′ and 5′-ATACTCCACGGGCTGGGTCTCC-3′; *sN3*∶5′-ACGTGAACGCCAACACCAAGC-3′and 5′-ACACGCCCATGAAGATGTTCCAC-3′. The PCR conditions were as follows: 94°C for 2 min, 35 cycles of 94°C for 30 s, 60°C for 30 s, and 72°C for 30 s, followed by a final extension period of 72°C for 10 min. The amplified products were subjected to electrophoresis on a 2% agarose gel. As a control, beta-actin gene was also amplified.

### Lipid Analysis

The fatty acid composition of total cellular lipids was analyzed as following: an aliquot of cell pellet homogenate in a micro reaction vessel (Supelco) was mixed with 1 ml of 14% BF3/MeOH reagent containing 0.005% butylated hydroxytoluene (as antioxidant). After blanketed with nitrogen, the mixture was heated at 95–100°C for 1 hour, then cooled to room temperature and methyl esters were extracted in the hexane phase following addition of 1 ml H_2_O and 1 ml of hexane. The samples were centrifuged for 5 minutes at 3000 rpm, then the upper hexane layer was removed and concentrated under nitrogen. Fatty acid methyl esters were quantified with GC-MS by using an HP-5890 Series II gas chromatograph equipped with an Omegawax 250 capillary column (Supelco, Bellefonte, PA) attached to an HP-5971 mass spectrometer. The oven program was maintained initially at 150°C for 2 min, then ramped to 200°C at 10°C/min and held for 4 min, ramped again at 5°C/min to 240°C, held for 3 min, and finally ramped to 270°C at 10°C/min and maintained for 5 min. Peaks were identified by comparison with fatty acid standards (Sigma), then area and percentage for each resolved peak were analyzed.

### Statistical Analysis

Statistical analysis was performed with SPSS version 18.0 for Windows using one-way ANOVA followed by LSD comparison test for posthoc analyses. Results were expressed as the mean ± SD. A p-value of <0.05 was considered to be statistically significant.

## Results

**Figure 1:**
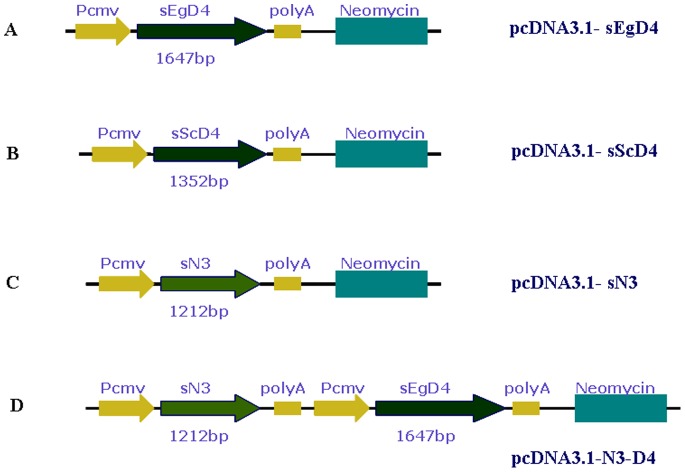
Construction of Recombinant plasmids for cell transfections. All synthetics of candidate genes were inserted into the vector pcDNA3.1^−^ by EcoR I /HindIII restriction sites in (A), (B) and (C). The double gene expression vector pcDNA3.1-N3D4 (D) was constructed based on pcDNA3.1-D4, with the insertion of pCMV-sN3-polyA DNA fragment by Bgl II/Mlu I restriction sites.

**Figure 2:**
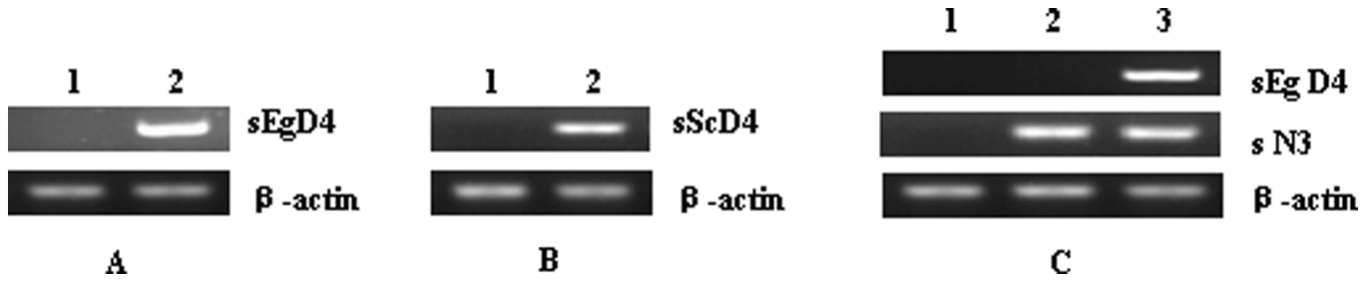
*sEGD4*, *sScD4* and *sN3* transcripts in transfected cells were analyzed by RT-PCR. (A) No. 1 and No. 2 were cells transfected with *EGFP* and *sEGD4* respectively; (B) No. 1 and No. 2 were cells transfected with *EGFP* and *sScD4* respectively; (C) No. 1, No. 2 and No. 3 were cells transfected with *EGFP*, *sN3* and *N3D4* respectively.

**Figure 3:**
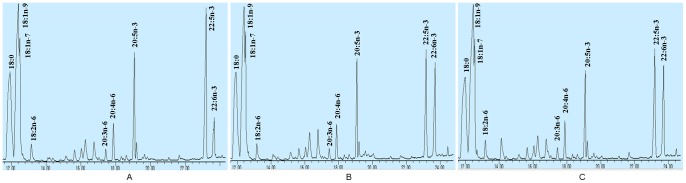
Partial gas chromatograph traces showing fatty acid profiles of total cellular lipids extracted from the control cells infected with *EGFP* (A), the cells infected with *sEgD4* (B) and the cells infected with *sScD4* (C).

The two Δ4 desaturase genes, *sEgD4* and *sScD4*, were successfully synthesized (with genetic codons optimized). Their gene expression vectors, pcDNA3.1-sEgD4 and pcDNA3.1-sScD4, were constructed (Fig. 1A and B). To test whether pcDNA3.1-sEgD4 and pcDNA3.1-sScD4 could be expressed in mammal cells, the vectors were introduced into human embryonic kidney (HEK) 293 cells by using liposome transfection method. DPA were added to the cell culture medium for 3 days. The RT-PCR results showed that the mRNA from these two synthetic genes could be greatly transcribed, while the cells transfected with the control plasmid pcDNA3.1-EGFP, which carried the enhanced green fluorescent protein as reporter gene, detected neither *sEgD4* mRNA nor *sScD4* mRNA (Fig. 2A and B). The transient transfections of pcDNA3.1-sEgD4 and pcDNA3.1-sScD4 in HEK 293 cells confirmed both genes could directly and efficiently convert DPA to DHA, as shown in Fig. 3. Both *sEgD4* and *sScD4* expression products had similar enzymic activity and conversion rate (Table 1).

**Table 1:** PUFA composition of total cellular lipids from the HEK293 cells transfected with EGFP, sEgD4 or sScD4 genes.

Mol % offatty acids	EGFP	sEgD4	sScD4
18∶2n-6	5.28±0.41	4.66±0.61	4.07±0.26
20∶3n-6	3.99±0.38	3.16±0.35	3.01±0.21
20∶4n-6	9.21±0.69	8.22±0.52	8.18±0.53
20∶5n-3	24.61±0.61^a^	24.18±0.59^a^	21.09±0.79^b^
22∶5n-3	44.17±1.12^a^	31.84±0.86^c^	34.86±0.97^b^
22∶6n-3	12.72±0.60^b^	27.92±0.74^a^	28.62±1.04^a^
DHA/DPA ratio	0.29±0.03^b^	0.88±0.05^a^	0.83±0.05^a^

Values are means of three measurements. Values for each fatty acid with the same superscript do not differ significantly (P<0.05) between control (EGFP), sEgD4 and sScD4.

In order to test whether the Δ4 desaturase genes can coorperate with *fat-1* gene in mammalian cells to convert DPA (one of the products catalyzed by *fat-1* ) to DHA, we constructed the *fat-1* gene expression vector, pcDNA3.1-sN3 and a double gene expression vector, pcDNA3.1-N3D4 (Fig. 1,C and D), for the expression of Δ4 and Δ15 desaturase activities in the same vector. The *sEgD4* gene was chosen for the construction of this double gene expression vector for it seemed to convert a little more DHA from DPA than *sScD4* (though it was not significant statistically). The vectors pcDNA3.1-sN3 and pcDNA3.1-N3D4 were transferred into NHK293 cells together with pcDNA3.1-EGFP as a control. Fatty acid substrates, 10 µM 18∶2*n*-6 and 10 µM 20∶4*n*-6, were added to the cell culture medium for 3 days. RT-PCR was performed to analyze the transcription levels of the introduced genes in transgenic cells. As shown in Fig. 2C, Δ15 desaturase mRNA were highly expressed both in pcDNA3.1-sN3 and pcDNA3.1-sN3D4 transfected cells, and Δ4 desaturase mRNA were highly expressed only in pcDNA3.1-sN3D4 transfected cells, while pcDNA3.1-EGFP transfected cells detected no Δ15 desaturase mRNA, and both pcDNA3.1-EGFP transfected cells and pcDNA3.1-sN3 transfected cells detected no Δ4 desaturase mRNA.

**Figure 4:**
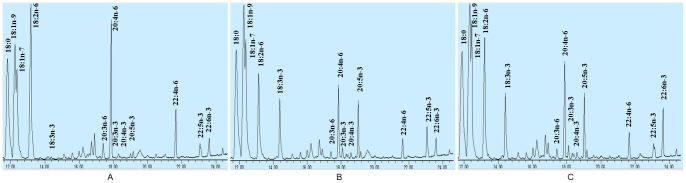
Partial gas chromatograph traces showing fatty acid profiles of total cellular lipids extracted from the control cells infected with *EGFP* (A), the cells infected with *sN3* (B) and the cells infected with *N3D4* (C).

Fatty acid composition of total cellular lipids extracted from the transfected cells was analyzed by Gas Chromatography-Mass Spectrometer (GC-MS). As shown in Fig. 4, the fatty acid profiles were remarkably different between the control cells transfected with pcDNA3.1-EGFP and the cells transfected with pcDNA3.1-sN3 and the cells transfected with pcDNA3.1-sN3D4. In the cells expressing the Δ15 desaturase, most of n-6 fatty acids were converted notably to the corresponding *n*-3 fatty acids: 18∶2*n*-6 to 18∶3*n*-3, 20∶3*n*-6 to 20∶4*n*-3, 20∶4*n*-6 to 20∶5*n*-3, and 22∶4*n*-6 to 22∶5*n*-3. However, in the cells expressing the Δ15 desaturase, the level of DHA obviously did not change, indicating that there was no fatty acid substrate that could be converted to DHA. In the cells expressing both the Δ15 desaturase and the Δ4 desaturase, the n-6 fatty acids were also converted notably to the corresponding *n*-3 fatty acids similarly as in the cells expressing the Δ15 desaturase, but the main difference was that DPA was reduced, while DHA was increased. As a result, the fatty acid composition of the cells expressing both the Δ15 desaturase and the Δ4 desaturase was more balanced compared with that of the cells expressing only the Δ15 desaturase. The ratio of *n*-6/*n*-3 was reduced from 8.76 in the control cells to 1.46 in the cells expressing the Δ15 and Δ4 fatty acid desaturases(Table 2).

**Table 2:** PUFA composition of total cellular lipids from the HEK293 cells transfected with *EGFP*, *sN3* or *sN3D4.*

Mol % offatty acids	EGFP	sN3	sN3D4
n-6 PUFAs			
18∶2n-6	50.52±1.29^a^	32.99±1.13^b^	35.47±0.92^b^
20∶4n-6	28.92±0.87^a^	17.45±0.64^b^	18.77±0.71^b^
22∶4n-6	10.30±0.59^a^	4.87±0.44^b^	5.22±0.51^b^
Total	89.74±1.41^a^	55.31±0.96^c^	59.46±0.99^b^
n-3 PUFAs			
18∶3n-3	0.64±0.03^c^	19.48±0.31^a^	16.14±0.27^b^
20∶5n-3	1.41±0.08^b^	11.86±0.22^a^	10.99±0.18^a^
22∶5n-3	3.92±0.11^b^	8.84±0.20^a^	4.12±0.13^b^
22∶6n-3	4.27±0.18^b^	4.61±0.16^b^	9.27±0.25^a^
Total	10.24±0.30^c^	44.79±1.07^a^	40.52±0.91^b^
n-6/n-3 ratio	8.76±0.27^a^	1.23±0.06^b^	1.46±0.07^b^

Values are means of three measurements. Values for each fatty acid with the same superscript do not differ significantly (P<0.05) between control (EGFP), sN3 and N3D4.

## Discussion

In this paper, we firstly reported the transgenic expression and enzymatic activity of two non-mammalian Δ4 desaturases in mammalian cells, as well as one of the Δ4 desaturases coorperate with Δ15 desaturase to convert DPA (a catalyzed product of Δ15 desaturase) to DHA in the transfected cells expressing the two types of desaturases.

In nature, the synthesis of DHA are very different among microorganisms, plants, and animals. The production of DHA in marine bacteria, such as *Shewanella sp*., *Vibrio* sp., and *Photobacterium profundum*, relies on polyketide synthase (PKS) systems, which are encoded by large gene clusters of about 30 kb [22]–[26]. LCPUFA production via this pathway might not be restricted to prokaryotes, for the oleaginous fungus *Schizochytrium sp*. has been shown to possess a gene cluster with significant similarity to the bacterial PKS systems [27]. Another strategy relies on the alternating action of desaturases and elongases, requiring fatty acid substrates such as linoleic acid and α-linolenic acid to start with. They constitute the ω- 6 and ω-3-pathways for polyunsaturated fatty acid biosynthesis. Besides the main routes leading to arachidonic acid and DHA, another route in different organisms makes use of a Δ9-elongase and a Δ8-desaturase [28]–[30]. It seemed that the PKS systems are more efficient in production of DHA than the ω- 6 and ω-3-pathways. Mammals possess the desaturases (such as Δ6 and Δ5) and elongases (Δ6, Δ5 and Δ7) needed in ω- 6 and ω-3-pathways, but they synthesized only very limited DHA in vivo from other common dietary n-3 PUFAs such as a-linolenic acid (ALA, 18∶3n-3). Some lower organisms (utilizing the PKS systems) such as *Thraustochytrium* sp., *Schizochytrium* sp., and the dinoflagellate *Crypthecodinium cohnii* accumulate as much as 50% DHA in their fatty acids [31]–[33]. However, the fact was more complex than this, because the cloning of a Δ5- and a Δ4-fatty acid desaturase from *Thraustochytrium* sp indicates both the PKS systems and the desaturases/elongases pathways might operate simultaneously in this organism [20]. The alga *E. gracilis* differs from the above-mentioned organisms by the fact that it accumulates only minor proportions of DHA (about 2% of total fatty acids), while it displays a wide range of different fatty acids with significant proportions of C16–C22 PUFAs [34]. The cloning of a Δ8-desaturase and a Δ4-desaturase from *E. gracilis* may be seen as additional evidence for the presence of a LCPUFAs biosynthetic pathway that involves desaturases and elongases [18], [28]. Interestingly, the *E. gracilis* Δ4-desaturase was as efficient at desaturating exogenously supplied DPA at the Δ4-position to produce DHA as *Thraustochytrium* sp. When heterologously expressed in yeast, the *E. gracilis* Δ4-desaturase converted about 29.7% DPA to DHA [18]. Coincidentally, a vertebrate fatty acid desaturase with Δ4 activity was cloned from *S. canaliculatus*, and also demonstrated that it conferred on the transgenic yeast the ability to convert 23% DPA to DHA [21]. As Δ6 and Δ5 desaturase activities were also reported in *S. canaliculatus*, it showed that such a teleost fish utilized the similar mechanism as *E. gracilis* for the production of DHA and other LCPUFAs. According to this, we thought the Δ4-desaturases from *E. gracilis* and *S. canaliculatus* was more suitable for the heterologous expression in mammals to convert DPA to DHA, for mammals possess similar PUFAs (e.g. 18C–22C) substrates for production of DHA as in both organisms. This paper has clearly demonstrated that, when heterologously expressed in mammalian cells(HEK 293), both the *E. gracilis* Δ4-desaturase and *S. canaliculatus* Δ4-desaturase can efficiently convert suplied DPA into DHA. What’s more important is that the proportions of DPA converted to DHA were about 37.5% in HEK 293 cells expressing *E. gracilis* Δ4-desaturase and 36.3% in HEK 293 cells expressing *S. canaliculatus* Δ4-desaturase, which is even higher than that in transgenic yeast expressing these two desaturases respectively. This indicates that the mammalian cells or even mammals are likely to accept these Δ4-desaturases activities from lower organisms.

As mentioned above, mammals synthesized only very limited DHA in vivo from it’s potential substrate PUFAs. Even the heterologous expression of the Δ15 desaturase in mammalian cells did not significantly increase the level of DHA [14]. This result clearly shows the inefficiency of the Sprecher pathway in DHA production. As shown either in Kang’s report [14] or in this study, the heterologous expression of the Δ15 desaturase in mammalian cells converted 22∶4n-6 to DPA almost as efficiently as it converted LA to ALA and ARA to EPA. This led to a relatively high proportion of DPA accumulated, but the DPA almost completely could not be converted to DHA through the Sprecher pathway. Our study coupled the Δ4 desaturase with Δ15 desaturase in the transgenic HEK293 cell, which successfully and efficiently converted the DPA to DHA. In other words, DPA formed by Δ15 desaturase activity from 22∶4n-6 was converted to DHA by Δ4 desaturase activity. The rate of DHA synthesis could be faster in the more direct Δ4 pathway that only requires endoplasmic reticulum, whereas the Sprecher pathway also involves peroxisomes, translocation of PUFA intermediates and limited fatty acid oxidation, a catabolic step. Some researchers thought the inefficiency of the Sprecher pathway in DHA production was due to Δ6 desaturase, the rate limiting enzyme in the PUFAs biosynthetic pathway – both at the initial steps of the pathway and at the terminal steps of the pathway (Sprecher pathway). However, another result in our experiment demonstrated the co-overexpression of Δ6/Δ5 desaturases in mammalian cells only led to high-level production of DPA, without obvious increase of DHA (unpublished data). Therefore, it seemed that all of the steps in Sprecher pathway together led to the inefficient DHA generation. The introduction of Δ4 desaturase into mammalian cells avoided these steps. Undoubtedly, this is a perfect combination of the Δ15/Δ4 desaturases for the production of high level of DHA in mammalian cells (very likely in mammals), as well as the dramatic reduction of the ratio of n-6/n-3 PUFAs.

Modern diets are high in saturated fatty acids, and low in unsaturated fatty acids [35], [36]. Among unsaturated fatty acids, the diets are high in n-6 fatty acids and low in n-3 fatty acids. It was reported that the average US intake of linoleic acid (LA) is 14.8 g/day, while arachidonic acid (ARA) is consumed at the amount of 0.15 g/day. The total intake of n-3 FAs in the US is 1.6 g/day. Of this, α-linolenic acid (ALA) accounts for 1.4 g/day and only 0.1–0.2 g/day comes from eicosapentaenoic acid (EPA) and docosahexaenoic acid (DHA) [17], [20], [37]. However, for most population in the whole world, their intake of fatty acids are nearly like this because of the extensive consumption of meat products (high in saturated fat) and vegetable oil (mostly high in LA). From this we see that ARA, EPA and DHA are the LCPUFAs that are most likely to be insufficient in today’s diets. Of the three LCPUFAs, DHA is more difficult than ARA and EPA to be accumulated from the biosynthesis pathways in mammals. To some extent, DHA is the PUFA whose intake should be elevated through diets. Our findings as presented in this paper provided a potential for land animals or livestock to produce high level of DHA as well as EPA and ARA (with balanced n-6/n-3 PUFAs). This will greatly increase the availability of these important LC-PUFAs for most people across the world without altering their dietary habits. After all, today’s dietary DHA and EPA supplements mainly come from marine products such as encapsulated fish oil, which may not be suitable for lifelong daily consumption. Furthermore, the decline in marine fish stocks and the potential contamination of marine products with mercury and other chemicals call for land-based dietary sources of LC-PUFAs (DHA, EPA and ARA).

In conclusion, this work has demonstrated that the heterologous expression of the two Δ4-desaturases in mammalian cells can still efficiently function, directly converting DPA into DHA, and conferring on mammalian cells the ability to produce high level of DHA. Our findings showed that the cooperation of the *E. gracilis* Δ4-desaturase with *C. elegans* Δ15-desaturase in transgenic HEK293 cells made a more desirable fatty acid composition – with drastically reduced n-6/n-3 PUFAs ratio and high level of DHA as well as EPA and ARA. Our data provides a basis for potential applications of the gene constructs for expression of Δ15/Δ4-desaturases in transgenic designer livestock with desirable fatty acid profile. We believe that such livestock will bring benefit to mankind–if safty of transgenic animal products is ensured.
